# Endothelin-1 axes in the framework of predictive, preventive and personalised (3P) medicine

**DOI:** 10.1007/s13167-021-00248-z

**Published:** 2021-08-04

**Authors:** Adriana Torres Crigna, Barbara Link, Marek Samec, Frank A. Giordano, Peter Kubatka, Olga Golubnitschaja

**Affiliations:** 1 Department of Radiation Oncology, University Hospital Bonn, Rheinische Friedrich-Wilhelms-Universität Bonn, Bonn, Germany; 2 Clinic of Obstetrics and Gynecology, Jessenius Faculty of Medicine, Comenius University in Bratislava, 036 01 Martin, Slovakia; 3 Department of Medical Biology, Jessenius Faculty of Medicine, Comenius University in Bratislava, 036 01 Martin, Slovakia; 4 Predictive, Preventive and Personalised (3P) Medicine, Department of Radiation Oncology, University Hospital Bonn, Rheinische Friedrich-Wilhelms-Universität Bonn, Bonn, Germany

**Keywords:** Predictive preventive personalised medicine (PPPM/3PM), Endothelin, ET-1, Endothelin axis, Vasoconstriction, Vasodilation, Vasospasm, Nitric oxide, Stroke, Female and male health, Mental health, Lifestyle, Stress reaction, Sense regulation, Wound healing, Pregnancy, Embryonic development, Pain sensitivity, Drug sensitivity, Treatment target, Suboptimal health, Ageing, Metabolic impairments, Cardiovascular disease, Neurodegeneration, Cancer, COVID-19, Individual outcomes

## Abstract

Endothelin-1 (ET-1) is involved in the regulation of a myriad of processes highly relevant for physical and mental well-being; female and male health; in the modulation of senses, pain, stress reactions and drug sensitivity as well as healing processes, amongst others. Shifted ET-1 homeostasis may influence and predict the development and progression of suboptimal health conditions, metabolic impairments with cascading complications, ageing and related pathologies, cardiovascular diseases, neurodegenerative pathologies, aggressive malignancies, modulating, therefore, individual outcomes of both non-communicable and infectious diseases such as COVID-19. This article provides an in-depth analysis of the involvement of ET-1 and related regulatory pathways in physiological and pathophysiological processes and estimates its capacity as

- a predictor of ageing and related pathologies,
- a sensor of lifestyle quality and progression of suboptimal health conditions to diseases for their targeted prevention
- and as a potent target for cost-effective treatments tailored to the person.

## Preamble

Endothelin (ET) is involved in the regulation and performance of a myriad of processes, which physiologically occur in a healthy human body. To them belong:

- Maintaining versus diminishing physical well-being [1, 2]
- Regulation of stress reactions and mental health: chronic as well as episodic psychosocial factors provoke social, environmental and emotional stress reactions; elevated plasma endothelin-1 (ET-1) influences individual differences in autonomic and hemodynamic responses to stress [3, 4]
- Female and male health [5–8]
- Pregnancy and embryonic development [9–12]
- Wound healing [13–16]
- Regulation of senses [17–20]
  amongst others.
  On the other hand, shifted ET homeostasis may influence and predict the development and progression of:
- Suboptimal health conditions [1, 18–24]
- Ageing and related pathologies [5, 25–28]
- Vascular stiffness and ageing, cardiovascular diseases and “young” ischemic stroke [1, 29–35]
- Metabolic impairments with cascading complications [1, 36–38]
- Neurodegenerative disorders [39–42]
- Particularly aggressive subtypes of cancer such as metastasing breast and prostate malignancies [7, 20, 43–50]
- ET-1 axes are involved in thermoregulation and attenuate the heat loss, modulate pain and drug sensitivity [20, 51–55], therapy response [56–58] and individual outcomes of both non-communicable [59] and infectious diseases, such as during the current COVID-19 pandemic [60–63].

This article provides an in-depth analysis of the involvement of ET-1 and related regulatory pathways in physiological and pathophysiological processes and estimates its potential as the diagnostic, prognostic and treatment target in the framework of 3P medicine.

## Historical notes

Endothelins were first described in 1985 by Hickey et al. as factors with vasoconstrictor actions acquired from a culture of bovine aortic endothelial cells [64]. They were suggested to have a chemical composition similar to peptides, due to the abolishing activity of trypsin [65]. Thereafter, Yanagisawa et al. defined the structure of these constricting factors as a 21-amino acid peptide named endothelin (designated endothelin-1 or ET-1), from a porcine aortic endothelial cell culture [66].

Shortly after, a similar peptide family named sarafotoxins was discovered in the cardiotonic venom of snakes and was seen to have a similar sequence to ET-1 [67, 68]. In humans, two further endogenous isoforms of endothelins were described while analysing the gene encoding ET-1 (edn1): endothelin-2 (ET-2) and endothelin-3 (ET-3). All isoforms consist of 21 amino acid residues, yet possess differential expression subject to their tissue or cell of origin [69]. In fact, due to differences in affinities for the three isoforms, in the upcoming year, two G protein-coupled receptors were identified, namely endothelin A receptor (ETA) [70] and endothelin B receptor (ETB) [71], where ET-1 and ET-2 signal through ETA in a more potent manner than ET-3, while all three isoforms are equally efficient in receptor ETB [72]. In fact, endothelins are secreted by numerous different cells, such as endothelial cells (EC), vascular smooth muscle cells (VSMCs), fibroblasts, renal medulla, leukocytes and macrophages [72]. The objective of this review is to summarise endothelin mediation in normal physiology and focus on its role in the pathogenesis of a number of affections and diseases.

## Endothelin-1 axes: function, physiology and measurement

After the discovery of ET-1, permanently increasing interest for its potent and sustained vasoconstrictor action in the pharmaceutical industry and academia sectors has been monitored. Endothelin isoforms are synthesised by respective cell types and tissues in the human being and encoded by responsible genes located in different chromosomes (ET-1, chromosome 6 and ET-2 and ET-3, chromosomes 1 and 20 respectively) [69]. ET-1, the main and parent component of the endothelin family, is secreted by most cell types with an increased expression in vascular EC, smooth muscle cells, cardiac myocytes fibroblasts, podocytes, macrophages and fibroblasts [73–75]. ET-1 has been described to be a multifunctional peptide involved in many physiological (cell differentiation and growth), pathological processes (cancer development and inflammatory events) and cell functions [76]. It plays a crucial role in pulmonary physiology, autoimmune disorders, neurological function and fluid and electrolyte transport [66, 69, 77, 78].

Synthesis of the ET-1 bioactive 21-aa peptide is carried out in multiple stages by the encoding gene edn1 via a proteolytic pathway [79]. Human gene edn1 transcription generates an mRNA that consequently encodes a 212-aa prepro-ET-1 [66]. Once prepro-ET-1 enters the endoplasmic reticulum furin-like peptidase split prepro-ET-1 into a 38-aa inactive peptide intermediate named big ET-1 [80]. Lastly, big ET-1 is converted by the endothelin-converting enzyme (ECE) to form the active form of ET-1 [81, 82]. ET-2 and ET-3 are also constituted from their inactive Big ET by ECE. Various ECE human isoforms have been described at different subcellular locations, ECE-1a, ECE-1b, ECE-1c and ECE-1d [83]. ET-1 plasma and circulating typical ET levels in numerous species is ∼ 1 pM suggesting that upon standard physiological conditions, endothelins are not circulating hormones but truly operate as paracrine and autocrine factors [84, 85].

ET-1 bioavailability regulation is described to happen mainly at a transcriptional level [86–88]. Proper reactivity of edn1 to stimuli is required for a correct regulation of ET-1 expression within the systems of the human body. In fact, modifications in edn1 expression or genetic polymorphisms have described alteration and pathogenesis of numerous diseases such as diabetic retinopathy, cancer, heart failure, cardiomyopathy or asthma [89–93]. ET elevated circulating levels are often related to cardiovascular diseases (CVD) such as ischaemic heart disease, hypertension, chronic heart failure, ischaemic heart disease or pulmonary hypertension. Nevertheless, increased ET levels have also been reported in non-CVD [85]. An enhancement of ET-1 mRNA expression has been reported in numerous cells by transforming growth factor-beta (TGF-β), interleukins, insulin, angiotensin II (AngII), tumour necrosis factor-alpha (TNF-α) and norepinephrine [66, 94, 95]. In EC, an upregulation of ET-1 mRNA is exerted by hypocapnia and downregulated by hypoxia [96].

As aforementioned, ET isoforms and ET receptors are distributed within a spectrum of tissues and by a variety of cells; hence, their interaction induces signal cascades promoting multifunctional outcomes. In fact, endothelium-released ET-1 (mainly secreted to the basal face) interacts via ETA with the smooth muscle, prompting vasoconstriction. In contrast, ET-1 coupling with ETB receptor regulates the secretion of relaxing factors such as prostacyclin (PGl2) and nitric oxide (NO) on neighbouring EC, inducing relaxation of smooth muscles [85]. Data suggests that endogenic ET-1 regulates peripheral blood flow via smooth muscle ETA, while diminution in blood pressure (BP) is regulated by endothelial cell ETB receptor [85, 97–100]. ET-1-ETA system has been described to be of importance in the cardiac and cranial neural crest, whereas ET-3-ETB in enteric neuron and melanocyte development [101, 102].

## Characterisation of ET subtypes

ET-2 differs from ET-1 in humans and other mammals namely cats, dogs, cattle and monkeys by two amino acids, Trp^6^ and Leu^7^. Synthesis of ET-2 has been described to be comparable to that of ET-1. Edn2 gene is transcripted into peproendothelin-2 (Prepro-ET-2) which is then cleaved by a furin into Big ET-2. Then, ECE-1 and ECE-2 convert Big ET-2 into mature peptide ET-2 [82]. Gardiner et al. reported in an in vivo study how Big ET-2 was transformed to exert cardiovascular effects similarly to Big ET-1 [103]. However, there is still some controversy regarding the efficiency of conversion of Big ET-2 by ECE, despite sharing an identical cleavable bond with ET-1; Big ET-2 conversion rate by ECE-1 and ECE-2 was respectively 5–7 and 7–10% as rapid as Big ET-1 transition [82]. A quantitative RT-PCR study on rats revealed ET-1 mRNA expression in all 16 analysed organs, though only detected ET-2 expression in lung, ovary, heart, stomach and intestine, being ET-2 distribution more organ restricted [104]. Increased levels were reported in ovary and in all intestine regions (duodenum, jejunum, ileum, colon and rectum) [105, 106]. Medulla oblongata and pituitary glands presented higher ET-2 mRNA levels than ET-1, though most brain areas (cerebellum and cerebrum) had low or undetectable levels of ET-2 [107].

ET-2 is synthesised in spectra of human tissues: their mRNA and/or peptide have been identified in the heart, lung, kidney, vasculature, intestine and ovaries, promoting intestinal contraction, ovulation, thermoregulation and lung alveolarisation [108–112]. Big ET-2 presented greater levels (∼ 2 pmol/l) than Big ET-1 in human plasma [113, 114]. Nevertheless, ET-2 concentration (∼ 0.9 pmol/l) is lower than ET-1 levels [115]. Both in mice and rats, ET-2 differs from ET-1 by three amino acids (Asn^4^, Trp^6^ and Leu^7^) and is termed vasoactive intestinal contractor due to its original identification in contracting mice ileum [116].

ET-2 presents distinct physiological and pathophysiological properties than ET-1. In fact, removal of the ET-2 gene in mice translates into a phenotype with severe hypothermia, growth deceleration, hypoglycemia and ketonemia [117]. ET-2 gene selective deletion in epithelial cells prompted great changes in mice lung morphology leading to low blood oxygen and elevated carbon dioxide levels. ET-2 function  is exerted in a paracrine manner, being ET-2 mRNA present in epithelial cells and receptor mRNA in the mesenchyme. Furthermore, ET-2 appears to have a significant role in ovarian physiology [117].

Many studies have emphasised an important role of ET-1 as a follicular development and luteal phase regulator [118–120]; however, ET-2 expression in ovaries is in fact higher than ET-1 [121]. Both ET-2 and ECE-1 were transitorily expressed during ovulation in rat ovaries [122]. Palanisamy et al. reported ET-2 expression in the granulosa cells in pre-ovulatory follicles. After superovulation induction in mice, they observed a drastic enhancement of ET-2 mRNA expression 11 h after, coinciding with follicular breach [108].

ET-2 also acts as a chemokine, being a chemoattractant for neutrophils even at low levels [123]. Furthermore, ET-2 stimulated macrophage chemotaxis via ETB receptor through the MAPK pathway, displaying similar activity as CCL2 inflammatory chemokine [124].

ET-3 differs in 6 amino acids within the N-terminal loop when compared to ET-1 and ET-2, which confers ET-3 with selectivity for ETB receptor [125]. The ET-3 synthetic pathway is primarily similar to that of ET-1 and ET-2. The transcription product of the edn3 gene, peproendothelin-3 (Prepro-ET-3), is cleaved by the furin enzyme into the inactive Big ET-3. The capacity of ECE-1 and ECE-2 to be able to cleave Big ET-3 has been argued. Data report a reduced conversion rate of Big ET-3 (1–3 and 4–9% respectively) when compared to Big ET-1 [82]. Nevertheless, Big ET-3 induces vasoconstriction in vivo, suggesting the existence of cleaving enzymes [103, 126]. Indeed, a zinc-dependent endopeptidase with a similar ECE-1 sequence, Kell, effectively cleaved Big ET-3, proposing an alternative synthetic enzyme for ET-3 [127, 128].

ET-3 is expressed in melanocytes, intestinal epithelial cells, renal tubular epithelial cells, placenta, melanocytes and brain neurons promoting the secretion of anti-inflammatory and vasodilating agents (particularly PGl2 and NO) [74, 129]. Furthermore, ET-3 has been detected in the heart, endometrium, brain and pituitary glands, where its levels were greater than ET-1 [130]. In human plasma, ET-3 was detectable at low ratios of ∼ 0.3 pmol/l, as well as Big ET-3 at ∼ 6 pmol/l [113]. Human EC are unable to synthesise ET-3, thereby inactive circulating Big ET-3 may be originated in the adrenal glands [131]. Elevated concentration of immunoreactive ET-3 has been found in the lungs, brain, intestine and pituitary gland of rats [132]. Prepro-ET-3 mRNA was detected in the submandibular gland, kidney, eye, stomach and spleen rat tissues [133], while Big ET-3 was seen in mast cells and macrophages within the gastrointestinal tract of rats [134]. Significant increases in Big ET-3 concentrations have been reported in haemodialysis patients (along with Big ET-1 and -2), even if their active peptide levels were moderately elevated [135]. Nevertheless, concentration changes related to disease have not been broadly researched.

## Balanced release of ET-1 and nitric oxide is crucial for health protection

NO is produced in the arterial wall and is one of the most effective vasodilator molecules, which arbitrate endothelium-dependent relaxation [136]. NADPH-dependent NO synthase (NOS) generates NO through an enzymatic conversion of l-arginine to l-citrulline [137]. NOS3 constitutive expression promotes NO production by the endothelium. NO functionality is extensive, due to which it acts as the main endothelium-derived relaxing factor, in order to maintain vascular homeostasis [136]. Endothelial cell-produced factors, ET-1 and NO, exhibit opposing actions on smooth muscle cell contraction; however, when balanced, they regulate local vascular tone [136]. Studies have revealed that NO and ET-1 can be mutually regulated in order to reach vascular tone homeostasis. In fact, stimulation of NO production in EC exerted a reduction of ET-1 expression and secretion [138]. Another study showed ET-1 induction of eNOS uncoupling [139] and blockage of ETA receptor mends NO-dependent vascular function in mice with atherosclerosis [140].

Endothelial dysfunction is characterised by a transition of the endothelium to a pro-inflammatory and reduced vasodilation state, in which there is an alteration of vasoactive factor (NO, ET-1) equilibrium [141]. Several studies have suggested that decreased NO concentrations and elevated vasoconstrictive ET-1 and serotonin (5-HT) might have an impact on high BP onset. In fact, imbalanced expression of ET-1 versus NO, together with the impaired 5-HT release, has been reported in essential arterial hypertension (EAH) [142]. EAH pathogenesis is associated with the endothelium and constitutes a social burden [142]. Aflyatumova et al. studied the association between the plasma ET-1, serum NO, serum 5-HT and platelet 5-HT and BP in adolescent males, along with their use as preclinical biomarkers for endothelial dysfunction and EAH [142]. Plasma ET-1 and serum 5-HT concentrations were increased regarding the controls in both paediatric pre-hypertension (PPH) and hypertension (PH) children, although PH presented significantly higher levels. NO serum levels on the other hand were higher in PPH than in PH patients, correlating negatively with BP values [142]. Thus, results suggest that ET-1, NO and 5-HT may be related to BP in adolescents and could potentially function as preclinical biomarkers of EAH. These results are in line with another study which described increased ET-1 plasma concentrations in hypertensive adolescents when compared to healthy subjects, combined with correlating ET-1 levels with systolic blood pressure (SBP) levels [143].

## Gender differences in ET-1 functionality

Numerous gender differences have been observed in the endothelin system; in fact, gonadal hormones play a crucial role in modulating gender-related disparities in the ET system [144]. Sex steroids are crucial for vascular homeostasis regulation [145]. In both experimental and human hypertension models, plasma ET-1 levels were significantly higher elevated in males than females [146–148]. Female plasma ET-1 levels oscillate throughout the menstrual cycle, being lowest during the luteal and follicular phase and higher in the menstrual phase [149]. Female sexual hormones inhibit ECE action along with ET-1 mRNA expression and diminish ETB receptor expression, thus restraining ET-1 levels [144]. In a bilateral ovariectomy study on female Sprague–Dawley rats, oestrogen replacement treatment decreased lung expression of ETA receptor and renal inner medullar expression of ETB receptor [150]. Another study with oestrogen administration in transexual male patients reported a decrease in ET-1 levels [146]. Furthermore, in pregnancy, ET-1 levels are also decreased, concordant with elevated oestrogen levels [151, 152]. The other significant female hormone, progesterone, was seen to inhibit in vitro ET-1 secretion both in resting and stimulated EC [153, 154].

Conversely, testosterone appears to increase ET-1 synthesis both in vitro and in vivo [144, 154, 155]. Testosterone also modulates vascular reactions to exogenous ET-1 [156]. However, a rat orchidectomy analysis revealed elevated ET-1, ETA and ETB receptors in the portal veins, suggesting that testosterone might act suppressing the ET system [157]. Erectile dysfunction may be an early indication of hypertension, being endothelial dysfunction often the link between these two conditions. Hypertension is strongly correlated with an increased release of procontracticle factors such as AngII, aldosterone and ET-1, which impacts vascular and erectile structures [6]. In the vasculature, ET-1 elicits reactive oxygen species (ROS), production by NADPH oxidase enzyme, which in turn releases ET-1, favouring a prohypertensive [158, 159]. Animal model studies reveal ET-1 to be essential in salt-sensitive hypertension-related ED [160, 161]. Furthermore, ET-1 generated Ca^2+^ influx alteration, prompting smooth muscle contraction in isolated penile tissue [162]. An in vitro study showed increased ET-1 Ca^2+^ influence in human smooth muscle cells derived from corpus cavernosum from patients with ED when matched with healthy subjects [163]. These data propose gonadal hormones to have tissue-specified effects on the ET system; hence, an altered equilibrium in menopausal women between estrogens and testosterone might worsen ET-1 vascular-related pathologies [5]. Regarding ET-1 sensitivity, males have been demonstrated to exert increased vasoconstriction than females in response to ET-1, which has been associated with the increased expression of ETA and ETB receptor in the saphenous veins and in the renal medulla in males compared to females [144, 164, 165]. Similarly, cultivated cerebral arteries from women presented a decreased vascular sensitivity to ET-1 in comparison to those from men [166]. Clinical and basic research studies have demonstrated that females have reduced ETA-mediated vasoconstriction than men [164, 167–169]. Females also display great vascular endothelial smooth muscle ETB receptor-mediated vessel enlargement, which counteracts the global constrictor vascular tone [169, 170]. In fact, a study in which an ETB receptor antagonist (BQ-788) was administered to healthy subjects revealed ETB receptor-mediated vasoconstriction in men and vasodilation in women [170]. This may indicate a leading role of ETB receptor function in women that in turn provide better outcomes in vascular-related disorders than in men. In a similar study, Stauffer et al. showed increased blood flow in men after administration with ETA receptor antagonist (BQ-123) when compared to women, indicating greater ETA receptor-related tonic constriction [169]. Indeed, specific patterns of ET-1 receptor subtype expression and localisation lead to gender disparities in vascular responsiveness to ET-1.

Gender difference within the ET-1 system has been also associated with vascular mediators in downstream cascades activated via ET receptor activation. Zimmerman et al. have reported gender differences concerning ET-1 mediation in oxidative stress generation, modulation of NO levels, inflammation induction and Ang II hypertension [171]. It has been hypothesised that the activation state of the ETB receptor limits ET-1-induced increase in plasma within oxidative stress in females compared to males [172]. Furthermore, female rats that lack ETB receptors have been demonstrated to have diminished renal NOS activity relative to males [173]. Also, intramedullar ET-1 infusion increased diuretic and natriuretic responses in female but not male rats, proposing larger ETA- and ETB-mediated increases in NO in females [174]. Further studies have described ET-1 effect on increasing calcium release from the inner medullar collecting duct cells in males as compared to females [175].

## ET-1, age and ageing: is differential diagnostic approach feasible?

In developed countries, ageing entails an increased prevalence of a variety of non-communicable and chronic diseases [176]. Amongst them are vascular diseases, myocardial infarction, stroke, heart failure, diabetes, obesity and cancer, with an increased incidence in the course of ageing [25, 176].

Ageing is related to particular changes in both the innate and adaptive immune systems, promoting an increased inflammatory milieu and oxidative stress leading to alterations in expression, release and increased ET-1 system activity [5, 26, 27]. Ageing along with chronic diseases are related to alterations in inflammatory processes and endothelial cell pathways, leading ultimately to disease development, being endothelial dysfunction an early result of vascular ageing [177]. In fact, healthy premenopausal women present a much favourable cardiovascular status than age-matched men associated with the vasoprotective effect of ovarian steroids. Hence, at menopause, an alteration in these circulating hormones increments women’s cardiovascular profile risk [178]. It is worth mentioning that most studies regarding ET-1 system activity in ageing have been investigated in male participants.

In human brachial arteries, an increase in ET-1 expression was related to ageing along with alterations in ETA-ETB ratios [28]. Ageing men displayed an enhancement in ET-1-dependent vasoconstrictor tone, being potentially alleviated with aerobic exercise [179]. Donato et al. revealed an increased ET-1 expression in vascular EC of healthy elderly men, related to a decreased endothelium-dependent dilation (EDD). In old mice, ET-1 receptor A signalling suppresssed EDD [28]. Indeed, elevated endogenous ET-1 mRNA and protein levels in plasma and vascular tissue have been associated with ageing in both female and male rats [180, 181]. In studies with aged mice and rats, they observed ET-1 dampened vasoconstriction (albeit increased ET-1 concentrations) potentially due to functional inability of endothelin receptor signalling pathways [179, 182, 183]. Furthermore, in aged male rats, ET-1 may prompt renal injury, which suggests an action of ET across vascular barriers into the kidney [184]. A study researching aged female mice hearts noted upregulation of ET-1 expression in in vitro cultures of late passage cardiac fibroblasts, along with downregulation of oestrogen receptor-α (ER-α), important negative ET-1. Comparable results with in vitro cultures of senescent cardiac fibroblasts were reported, observing also an increased expression of fibronectin and collagens, which suggests that ageing-related cardiac fibrosis is in part subject to ET-1 upregulation [185].

Other age-associated diseases such as glaucoma or age-related macular degeneration (AMD) presented increased ET-1 which suggested its role in the diagnosis and pathogenesis of AMD [186, 187]. Enhanced levels of ET-1 were found in AMD patients treated with angiogenesis inhibitors (bevacizumab), yet its implication has not yet been determined [188]. Glaucoma patients have revealed increased ET-1 circulating and intraocular levels and anterograde-retrograde axonal transport [189–193]. ET-1 levels were able to predict postsurgery intraocular pressure in patients undertaking primary open-angle glaucoma eye surgery [194].

## ET-1 relevance for endocrine status and multi-faceted hormonal regulation

Diseases related to physiological ageing leading to endocrine changes, such as menopause, are enhanced with age, presenting constraints of the arterial vascular bed, arterial hypertension, insulin resistance and diabetes [195–197].

In fact, as previously described, premenopausal women are more advantageous than age-matched men regarding the cardiovascular phenotype, largely on account of the vasoprotective role of female sex hormones [198]. However, significant hormonal alterations are associated with menopause such as decreased levels of progesterone and oestrogen [199, 200]. Furthermore, estradiol (E2) also declines while follicle-stimulating hormone elevates, increasing the androgen:oestrogen ratio in postmenopause women [201]. Together with the ageing process, this may have implications on vascular and endothelial function, transitioning to a high-risk cardiac profile [199, 200]. It is known that E2 affects NO production and secretion in a direct manner via ERα, which might promote endothelial dysfunction due to a decrease in NO accessibility [202]. In a study with postmenopausal women, they found lower expression of ERα receptors and eNOS in peripheral vein-derived endothelial cells in comparison to young subjects, which suggest flow-mediated vasodilation to be dependent on their expression [203]. This indicates that endothelial function deterioration is partially dependent on reduction of ERα expression and eNOS, in turn affecting NO secretion.

The endothelin system and ET-1 are crucial in vascular dysfunction pathogenesis and have been seen to be affected by ageing. In fact, studies have shown an increase of endothelin in plasma in postmenopausal women [204, 205] along with elderly men [28] in comparison with young adults. In a menopause rat model, they observed greater mesenteric artery reactivity to ET-1 precursor, Big ET-1, compared to young females, which improved with oestrogen treatment [206]. Moreover, in studies with ovariectomised rats, the treatment with oestrogen neutralises ET-1 expression, suggesting a vital role of oestrogen on ET-1 regulation [147, 168, 207]. In contrast, testosterone is known to elevate ET-1 expression in both in vivo and in vitro conditions, thereby modulating responses to external ET-1 [154, 156]. In a Brazilian study on postmenopausal women, they revealed a direct relation between ET-1 and testosterone levels in serum [208].

Indeed, in postmenopausal women, ET-1 promotes the deterioration of endothelial function. However, a study with ETB antagonist BQ-788 has demonstrated the involvement of ETB receptor in restoring vasodilation in ageing women [204]. Contrarily, in the same study, the ETA antagonist showed no changes in vasodilator capacity, suggesting the loss of endothelial function to be associated with a decline in ETB receptor-mediated dilation [204].

Furthermore, in other endocrine defects such as thyroid diseases (Hashimotos’s thyroditis, Graves’ disease), they have also detected increased ET-1 plasma levels when compared to healthy controls [209]. Other studies have also described elevated ET-1 plasma levels in Graves’ disease [210, 211]. Nevertheless, patients suffering from endemic goitre did not present increased ET-1 levels [209]. They failed to observe a relation between ET-1 and thyroid disease parameters (thyroxine, thyroid volume, thyroid-stimulating hormone) [209]. Letizia et al. also reported a lack of correlation between the ET-1 levels and thyroid hormones [211]. Another study has observed that thyrocytes from Graves’ disease patients were stimulated by ET-1 to proliferate to a higher extent than healthy subjects [210]. Molet et al. demonstrated that after 24-h treatment with beta chemokines (macrophage inflammatory protein 1-alpha and monocyte chemotactic protein 1), EC elevated ET-1 mRNA in vitro secretion [212]. Thus, beta chemokines are involved in inducing ET-1 release in autoimmune and inflammatory diseases namely, Graves’ disease and Hashimotos’s thyroiditis [209]. In a study with hyperthyroidism patients, they observed increased plasma ET-1 concentration as opposed to control subjects [213]. They further discovered serum triiodothyronine and free thyroxine to be positively correlated to plasma ET-1 levels, along with a decrease in ET-1 levels after hyperthyroidism treatment compared to pretreatment values [213].

ET-1 has also been described to regulate the development and secretion of the adrenal gland [214]. The involvement of the ET system was detected both in human and rat adrenal cortex and in vitro carcinoma cells [215, 216]. Preproendothelin-1, ECE-1 and ETA and ETB mRNA were found in normal rat and human adrenal cortex [214]. In vivo studies with ET-1 administration to rats and dogs revealed an increase in blood pressure and plasma aldosterone levels [217], speculating the influence of ET-1 on the adrenal cortex [217]. Furthermore, this was supported by increased plasma aldosterone levels due to subcutaneous ET-1 infusion in rats [218]. Altogether, the ET system has an important function in the regulation of adrenal cortex function and potential contribution in adrenal gland pathogenesis like Conn’s adenoma [214].

## ET-1 association with adverse health effects related to abnormal body weight

Studies investigating endothelin mediation in low BMI subjects are currently limited. However, an epidemiologic study aimed to determine the correlation between BMI (comprising low BMI) and endothelial dysfunction [38]. Physiological control of vascular tone needs for an equilibrium between vasoconstricting and vasodilating factors [219]. Indeed, endothelin-1 and AngII circulating levels were similarly increased in low BMI, normal, obese and extremely obese groups. Levels of oxidative stress were analogous in low BMI, normal and obese subjects, although significantly greater when compared to normal BMI. Thus, results suggest that besides obesity, low BMI may also present a risk factor for diminished endothelium-dependent vasodilation in subjects, as a result of reduced NO bioavailability, with potential CVD prevalence [38]. Moreover, subjects with abnormally low BMI (underweight) have reported an increased risk of breast cancer with a poor prognosis [220]. Ferri et al. have reported increased plasma ET-1 levels in lean patients with essential hypertension, hyperlipemia and glucose intolerance compared to normotensive or subjects lacking these metabolic disorders [221]. Fasting insulin levels in plasma were also correlated to elevated ET-1 plasma levels, referred to as a risk factor for hypertension [221]. Another study tested leptin vascular actions on ET-1 system and NO pathway balance in both lean and obese individuals and tested the effects of ETA receptor antagonist, BQ123 and NOS inhibitor, NG-monomethyl l-arginine (l-NMMA) [222]. Leptin circulating levels were significantly greater in obese patients than in lean subjects, as previously observed [223, 224]. After infusion of BQ123 and l-NMMA, hyperleptinemia lean subjects revealed enhanced vasodilator responsiveness and greater vasoconstrictor response to l-NMMA during ETA receptor antagonism [222]. These data suggest that in healthy subjects, leptin supports vascular homeostasis by balancing both the ET-1 system and NO pathway, whereas exogenous leptin actions are absent in hyperleptinemic patients with obesity-related metabolic syndrome (MetS) [222]. Besides, increased production of ET-1 vasoconstrictor protein, impaired NO-dependent endothelial function and oxidative stress have been related to increased abdominal fat and body mass index [225].

Subjects with elevated body mass index (BMI) (both overweight and obese) present a chronic metabolic disorder with a distinct detriment of their cardiovascular health along with increased morbidity and mortality [226, 227]. Characterised by an increased BP and an incidence of hypertension [228], this threat may result from endocrine and paracrine dysregulation and chronic inflammatory state that derive from irregularities in adipose tissue function [229]. This consequently elicits an instability amidst the NO pathway and ET-1 system resulting from a disruption in vascular homeostasis [229].

Endothelial dysfunction is marked by diminished NO levels in obesity as presented by Steinberg et al. [230]. In patients with elevated BMI, they observed a dampened increase in leg blood flow in reaction to intra-arterial delivery of muscarinic receptor agonist methacholine, compared to lean subjects [230]. These results were reproduced by several groups [231, 232]. A further study revealed an impairment of brachial artery flow-mediated dilation (FMD) in obese patients. This suggests obesity-related metabolic abnormalities to prompt brachial artery endothelial dysfunction [233]. Furthermore, Woo et al. reported an association between brachial artery EDD impairment and mild to moderate obesity [234, 235]. Nutrition adjustments have been seen to partially restore vascular abnormalities specifically when combined with physical activity [234].

Moreover, increased ET-1 vascular action in pathologic conditions such as obesity, hypertension, diabetes mellitus and insulin resistance lead to BP abnormalities [36, 37, 236, 237]. Levin et al. revealed a key role of ET-1 in the pathophysiology of vasomotor morbidities linked to the formation of atherosclerotic plaque and endothelial dysfunction [238]. This knowledge was supported by a study investigating ET-1 and NO system interaction in obese patients, where the addition of selective ETA blocker (BQ-123) compensated the limitation in endothelium-dependent vasodilation, confirming ET-1 contribution to abnormal vascular homeostasis in such patients [239]. Indeed, increased ET-1-mediated vasoconstriction in insulin-resistant conditions was identified in obesity and diabetes subjects [240]. Yoon et al. performed a study with obese patients, observing increased peripheral vasodilation in this group when compared to lean subjects. This revealed endothelin as a potent vasoconstrictor affecting vascular tone and diastolic blood pressure [228]. Another study investigated the relationship between elevated ET-1 activity in hypertensive subjects and increased body index. Increased vasodilator responses to ETA blockage in overweight and obese patients were seen when compared to lean hypertensive subjects [241]. This ETA-dependent vascular reaction in a hypertensive and increased BMI setting may suggest an increased vasoconstrictive response to ET-1 or an enhanced ET-1 synthesis at the receptor site [241]. In a study researching ET-1-mediated vasoconstrictive tone, they revealed a dampened forearm constrictor response to external ET-1 in obese and overweight patients in relation to lean subjects [242]. Moreover, selective ETA receptor blockage elicited an increased forearm vasodilator response in subjects with higher BMI [242]. Shortly, ET-1 vascular action increased in overweight and obesity along with diminished endothelium-dependent vasodilation which may lead to an enhanced risk of hypertension and atherosclerosis disease. Physical activity and lifestyle changes can indeed improve endothelial function and reduce selective markers of endothelial activation [243]. In fact, a study carried out with obese men revealed a direct correlation between the percentage of weight loss and decline in plasma ET-1 concentration upon the conclusion of a low-caloric diet, which may improve obesity-induced endothelial dysfunction [244].

## ET-1 as the trigger of pro-inflammatory pathways

Numerous studies have proven the involvement of ET-1 in vascular inflammatory processes. ET-1 has been described to be related to inflammatory responses through activation of transcription factors (i.e. NF-κB) and expression of pro-inflammatory cytokines such as IL-1, IL-6 and TNF-α [245], which in turn stimulate secretion of ET-1 [246]. In particular, TNF-α is known to enhance inflammatory responses by means of cytokine cascades and is involved in the pathogenesis of several diseases such as rheumathoid arthritis, sepsis, Crohn’s disease, diabetes and obesity [247]. TNF-α induces ET-1 secretion in vascular EC [248, 249]. Besides, activated T cells release of TNF-α and IFN-γ induce monocytes to produce ET-1 in human peripheral blood mononuclear cells [250]. Other studies claim T cell-secreted TNF-α, IFN-γ, IL-4 and IL-10 to generate distinct macrophage types [251, 252]. Studies have proven that ETA receptor antagonists, namely BQ1232, have a favourable impact on TNF-α levels [253], lowering their concentration in patients after bypass grafting [254]. TNF-α and IL-1β expressions were also inhibited in an oxidative stress lung rat model by BQ123 (Chen et al. 2010). ETB receptor antagonist studies display some conflicting results. Tonari et al. revealed inhibition of TNF-α expression by BQ788 antagonist in patients with optic nerve damage [255]. Nevertheless, another group stated no substantial reduction in TNF-α levels in rat hearts following BQ788 infusion [256].

Indeed, increased production of pro-inflammatory cytokines can trigger the production of prostaglandin (PG) in vascular endothelial and smooth muscle cells [257]. A number of pathways (NF-κB, cyclooxygenase (COX) and NADPH oxidase-dependent) have been seen to promote ROS production in different cell types [258–260]. COX is a key enzyme in PG synthesis [178], and studies have indicated that its expression can be induced by ET-1, together with prostaglandin E2 (PGE-2) release by NF-κB and MAPKs [257]. ET-1 stimulated surface expression of vascular adhesion molecule-1 (VCAM-1) in TNF-α-stimulated vascular EC. This ET-1-promoted increase may be due to TNF-α concomitant inhibitory action on endothelin-induced NO production [261]. A further study with hypertensive patients revealed stimulation of arterial VCAM-1 by ET-1 [262]. VCAM-1 along with intercellular adhesion molecules prompt the adhesion of inflammatory cells to the vascular surface, involved in atherosclerosis evolution [263]. Anggrahini et al. investigated the effects of ET-1 on vascular inflammation and neointima formation in an ET-1-knockout mice model. They observed a decrease of ET-1 adverse effects, suggesting its participation in vasoconstriction and inflammatory cell recruitment to the vessels [264]. Moreover, ROS are critical physiological molecules in vascular cells which overproduction leads to the development of atherosclerosis and endothelial dysfunction [265]. The increase in oxidative stress, monocyte/macrophage infiltration and decrease in high-density lipoproteins are effects exerted by ET-1, leading to the progression of atherosclerosis and aneurysms [266]. Furthermore, overexpression of ET-1 in the endothelium of atherosclerotic mice was marked by a reduction of endothelial signalling pathways in charge of endothelium-dependent relaxation [267]. ET-1 receptor antagonist might be beneficial in preventing numerous vascular-related diseases [268, 269].

## ET-1 functionality is linked to both physiologic and impaired healing

Chronic wounds (non-healing)/ulcers are wounds that have not completed the physiologic wound healing (WH) process in an effective manner, arresting usually at early inflammatory stages [13]. Some chronic wound features include neutrophil infiltration, extended inflammatory phase and consistent infections [270, 271]. Risk factors related to the chronic wound-deficient healing process are both modifiable (smoking, malnutrition, obesity, diabetes, alcohol consumption, CVD) and non-modifiable (genetic traits and ageing) [13]. Wound-associated pain comprises both physiologic and psychologic factors, involving also stress and anxiety [15]. The immune system might be compromised by cortisol overproduction, and catecholamine-induced poor tissue oxygenation affecting WH in a direct manner [15]. In fact, individuals suffering from Flammer syndrome present elevated ET-1 levels causing systemic hypoxia as a result of improper vasoconstriction or deficient vasodilation [18, 272]. Wound healing in the liver is an integrated process that involves remodelling, fibrogenesis and disruption of liver structure, and accumulation of extracellular proteins leads to the conversion of the liver to fibrotic and even cirrhotic state [14]. ET is of great importance for WH in the liver; in fact, ET-1 and -3 circulating levels were seen elevated both in cirrhosis patients and in preclinical hepatic WH models [273, 274]. Immunoreactive ET-1 levels in the liver might be related to liver disease severity [275]. Sinusoidal EC usually produce ET in a healthy liver, although following an injury, ET-1 synthesis transitions to hepatic stellate cells [276–279]. Furthermore, ET-1’s primary aims in the liver are stellate cells, which possess a greater number of ET receptors and enhance their binding sites upon activation [280–282]. Activation of stellate cells is exerted by numerous factors such as cytokines, chemokines, growth factors, oxidative stress and ET-1 [283, 284]. ET-1 also interacts with TGF-β in modulating hepatic stellate cell activation and increases TGF-β1 mRNA besides stimulating the release of TGF-β1 in these cells. Inhibition of ET signalling by the ET receptor antagonists has decreased hepatic fibrogenic response [284–287]. In a chronic liver injury model, bosentan was able to inhibit ET-1-induced fibrogenesis [284].

## ET-1 is involved in the regulation of senses

Olfactory-sensitive neurons and their axons are enveloped by glia-like cells called sustentacular cells, which help to sustain the olfactory mucosa (OM) structure and ionic integrity [17]. A group demonstrated that sustentacular cells were sensitive to ET-1 and thereby able to uncouple gap junctions [288, 289]. Moreover, endothelin has been described to inhibit gap junctional communication effectively in astrocytes [290]. ET receptors were also greatly expressed in the OM, being ETB receptor mostly expressed in the olfactory sensory neurons, while most non-neuronal cells mainly express ETA receptors [288, 289]. Another investigation found ET-1 acted as a neuroprotective for olfactory cells [291]). In a recent study, they evaluated if OM-produced ET-1 could have an effect on olfactory processing in young rats [17]. OM responses to odorant stimulation following local ET-1 application revealed no modification of response amplitude but slowed treatment recovery. The maternal odour recognition orientation test was decreased after ET-1 treatment, which overall suggests ET-1 olfactory response regulation only partly via gap junction uncoupling [17].

Relation between increased levels of ET-1 levels in blood serum in retinitis pigmentosa and FS has also been described [292]. In accordance, a case report described how a female patient suffering from FS presented significantly elevated serum endothelin levels [19]. ET-1 effect on PGE-2 increased production could potentially suppress the feeling of thirst in such individuals [19].

## ET-1 modulates pain and drug sensitivity

Endothelins contribute to numerous pain-related processes, namely, pain caused by cancer, inflammation or Sickle cell disease (SCD). In the central nervous system (CNS), administration of ET-1 intracerebroventricularly (i.c.v) was formerly reported to present antinociceptive actions, which suggests ET-1 involvement in pain transmission [293, 294]. In fact, in a study, they investigated the ET-1 mediation of antinociceptive actions in mice that received acute thermal pain testing by examining their threshold variations [51]. They confirmed dose-dependent antinociceptive action of ET-1 following i.c.v administration, suggesting that ET-1 effects implicate a descending pain inhibiting system. Moreover, they observed a blockage of antinociceptive events due to the ETA receptor antagonist, indicating it being mediated by ETA receptor activation [51].

In addition, ET-1 has been described to be overexpressed in breast carcinoma patients and is associated with poor prognosis, as well as displaying a strong link between both preoperative and postoperative pain sensitivity [20]. The younger age breast cancer patient cohort  might be at increased risk for elevated pain sensitivity [53]. In another study, ET-1 administration to study participants elicited dose-dependent effects regarding spontaneous pain and temperature perception [54]. Furthermore, in SCD patients and mouse models, ET-1 blood plasma levels have been described to be elevated, resulting in acute and chronic pain episodes. It is thought that ETA receptors support SCD-derived pain by primary sensory neuron NF-κB-triggered upregulation of Nav1.8 [55]. In a study, they investigated the mechanism by which ET-1/ETA receptors participate in SCD-associated pain. They showed that ET-1 and ETA receptor levels were elevated in the dorsal root ganglia of humanised mouse SCD models, but pharmacologic inhibition of ETA receptors (in primary sensory neurons) by ABT-627 [295] mitigated basal and post-hypoxia pain hypersensitivities [55]. These findings suggest the ETA receptor as a potential target for SCD pain management, although further clinical research must be performed.

ET-1/ET-1R axis activation gives cells the potential to exert changes in cell fate and accomplish deleterious features [296]. ET-1 expression has been detected in numerous malignancies such as in advanced tumoral contexts [297, 298], where elevated ET-1R indicated worsened prognosis [296]. Within tumours, ET-1 generates signals which induce pro-survival transcriptional answers, securing tumoral cells from cancer therapy-induced apoptosis [297, 299, 300]. Indeed, ovarian cancer (OC) patients have poor survival rates due to late diagnosis at clinical stages along with recurrence of the disease due to failure of platinum-based chemotherapy [301]. Chemotherapy resistance consists of adaptive signalling pathways which develop specific transcriptional profiles [302]. Platinum-resistant OC tumours have been seen to express greater levels of ETA receptor, being linked to worse disease prognosis [300]. It was revealed that the ET-1/ETA receptor axis hinders the yes-associated protein (YAP) pathway in platinum-resistant OC cells, which crosstalk hinders chemotherapy-induced apoptosis [52]. Tocci et al. effectively described co-therapy of ET-1 receptor antagonist and cisplatin to achieve sensibilisation of platinum-based therapy-resistant cells, reducing in turn their metastatic potential [52]. Conclusively, blocking the ET-1 receptor enhances platinum-induced apoptosis and hampers adaptive systems, constituting a favourable therapeutic approach to enhance OC patient drug sensitivity.

## ET-1 modulates stress reactions and mental health

Psychological social risk factors such as social environment (job or family stress, low socioeconomic status, detrimental life events) and emotional factors (depression, anxiety, exhaustion) have been known to strongly relate to a higher risk of CVD [3, 303]. In the INTERHEART study, they evaluated crucial standard CVD risk factors and a series of psychosocial factors (depression, financial stress) related to acute MI. Results revealed an odds ratio in women of 3.49 and 2.58 in men, independent of ethnicity and geographic origin [304]. Notably, these odd ratios were comparable to CVD risk factors such as diabetes, hypertension or smoking [305]. This suggests an overall contribution of psychosocial factors to an increased prevalence of CVD, being ET-1 balance disruption directly linked to these psychosocial-induced mechanisms [3]. Yammime et al. thoroughly reviewed studies dealing with the relation between these factors and found studies proving heterogeneous findings [3]. Studies on young men and women with CVD family history revealed ET-1 higher levels in African Americans compared to European Americans, following psychological challenges [306]. Sex differences in ET-1 stress reactivity were evaluated, observing significantly increased ET-1 plasma levels in males compared to females [307]. However, there was great variability in protocols used for ET-1 assessment, time of sample collection, laboratory detection procedures and the type and duration of mental/psychological challenges, making it challenging to draw clear conclusions [308–311]. Evidently, ET-1 measurements  have to be obtained at regular intervals during a post-stress recovery period in order to elucidate ET-1-specific kinetics [308]. Vascular dementia follows Alzheimer’s disease (AD) as the most common type of dementia worldwide, caused mainly by ischaemic or hemorrhagic cerebrovascular (CVS) disease; they cause cognitive detriment and neurodegeneration [312–314]. A recent study focused on examining whether a combination of Shenmayizhi formula and Ginkgo extract positively impacts mild to moderate VaD [4]. Serum indexes of vascular endothelial function, namely, ET-1, NO, VEGF and von Willebrand factor (vWF) were measured. Posttreatment serum ET-1 and vWF levels had decreased, whereas VEGF and NO levels increased in the SMYZF group, presenting more significant changes than the Ginkgo group [4]. This suggests that SMYZF administered with Ginkgo tablets may potentially improve vascular endothelial function, assisting with cognitive improvement and neurological functions in VaD patients. Other studies also described an association between vascular cognitive impairment severity and ET-1 levels [315, 316]. Furthermore, ET-1-induced vasoconstriction prompts cerebral ischaemia and hypoxia which in turn promotes dementia development [317].

## ET-1 and cardiovascular diseases

ET-1 levels have been reported to be elevated in a number of CVD such as acute myocardial infarction (AMI) [318], coronary artery disease, hypertension, atherosclerosis and congestive heart failure (CHF), amongst others [29]. On account of its vascular tone and contractive potential, ET-1 has been suggested to be involved with hypertension development in humans [29]. In murine models of hypertension, ET-1 levels were increased only if accelerated hypertension is displayed [319, 320]. Studies comparing ET-1 expression in blood vessels from deoxycorticosterone acetate (DOCA)–salt rats and normotensive Wistar–Kyoto (WKY) rats revealed larger amounts in the former, which indicated strain-related differences in ET-1 production, responsiveness or proportion of ETA and ETB vessel receptors [321]. Human studies also revealed an increase in BP after infusion of ET-1 to healthy subjects [322]. ET-1 blocking with bosentan led to a decrease in BP in hypertensive patients [323]; nevertheless, it also diminished BP in essential hypertension subjects with normal ET-1 levels [324], potentially indicating that plasma ET-1 levels do not show the real state of endothelin action.

Hypercholesterolemia is linked with elevated ET-1 levels in human tissues and plasma [325]. It has been hypothesised that ET-1 may be relevant for atherosclerosis formation at all stages, even at the outset [326, 327] seen that ET receptor blockade decreased premature atherosclerosis [328]. More recent animal studies have described how oxidised low-density lipoprotein prompt mRNA expression and release of ET-1 from EC, proposing a key role of ET-1 in atherosclerosis progression [329]. ET receptor antagonist was also able to hinder early atherosclerosis formation in hyperlipidemic hamsters [328]. In humans, there are many factors that can influence atherosclerosis such as hypertension, obesity, diabetes mellitus or hypercholesterolemia [330], and even cytokine release and inflammation may promote atherosclerosis [331]. Furthermore, ET-1 increased levels induce platelet-derived growth factor (PDGF), fibroblast growth factor, TGF-β1 and vascular adhesion molecules synthesis [332].

Ischaemic heart disease is the primary mortality cause worldwide [333]. In experimental models, coronary vessel constriction was induced by ET-1 [333], demonstrating that coronary vasosparm is related to ET-1 hyperactivity. ET-1 activation impeded NO-mediated dilation, which might promote vasospam in coronary arteries [334]. ET receptor antagonist extended rat long-term survival following AMI [335]. Coronary artery disease patients might eventually develop acute coronary syndrome (ACS) or AMI. In fact, AMI patients presented elevated plasma ET-1 levels [336] correlating with 1-year mortality [337]. Further studies have shown how endogenous ET-1 exerted vasoconstrictor on arteries, demonstrated by the increased coronary flow following ET-1 receptor blockade in both coronary artery diseased [338] and common coronary patients [339]. Patients with ACS presented enhanced myocardium and left ventricular tissue perfusion following selective ETA blocker therapy [340]. In accordance with these results, ETA and ETB receptor blockage evidenced vasodilation in coronary atherosclerosis patients [338].

The endothelin system is also involved in chronic heart failure pathophysiology, being ET-1 plasma levels higher in patients with CHF, and resting values almost twofold/threefold greater than in healthy subjects [341]. This raise is thought to be primarily due to an increased big ET-1 and ET-1 production [342], supported by the release by the lungs and myocardial cells [343]. CHF patients’ symptoms and hemodynamics have been correlated to circulating ET-1 levels [31], being big ET and ET-1 independent survival predictors [344, 345]. Other investigators also showed how plasma ET-1 in CHF was related to more severe disease [346].

Laboratory studies reveal that ET-1 might cause arrhythmic effects in CHF settings [33], besides being involved in cardiac remodelling via fibroblast activation, inflammation of the heart and activation by renin–angiotensin–aldosterone system stimulation [34, 347]. In fact, ECE inhibition promoted the inactivation of the renin–angiotensin–aldosterone system in the CHF milieu [348]. Human studies  prompted to clarify the functional link between CHF and ET system activation and progression to be a compensatory neuro-humoral adaptation [342, 344, 349], being patients with the highest ET-1 levels the ones with worse prognosis [344]. Blocking the ET system has turned into a main target for therapeutical treatments.

Preclinical studies with rats demonstrated the use of ERA, namely, BQ-123 to enhance myocardial function and viability [350, 351]. In humans, intravenous administration of BQ-123 [352] or bosentan [353] diminished systemic and pulmonary vascular resistance and BP. Although CHF treatment with ERA was promising, none of the four multicenter, controlled randomised clinical trials was prosperous [354]. Dhaun et al. thoroughly review the discontinued trials [355]. Moreover, acute heart failure portrayed similar results. Initial studies with ERA did in fact propose a haemodynamic advantage, ultimately showing no major effect [356–358]. Whether lower drug doses would have displayed beneficial effects, currently remains unknown [359]. Jankowich et al. have thoroughly reviewed the potential use of ET-1 as CVD prognosis on account of more personalised treatment schemes [30]. In stable angina patients, big ET-1 high levels were indicative of cardiovascular events such as non-fatal myocardial infarction and stroke, showing a diminished event-free survival [360]. ET-1 may also predict post-myocardial infarction phenomena, namely, the absence of tissue perfusion after percutaneous coronary intervention therapy or even mortality [361]. However, C-terminal proET-1 levels anticipated heart failure in high-risk cardiovascular subjects but not in low-risk patients [362]. Other studies of coronary revascularisation or cardiac catheterisation have also failed to determine cardiovascular-related events [363, 364]. Thus, even if ET-1 peptide levels may support the prediction of cardiovascular events in patients with stable coronary artery disease, this requires further study in multiple cohorts and populations in order for ET-1 to allow more personalised therapy regimens.

## ET-1 and Takotsubo syndrome

A group of Japanese cardiologists identified a disease characterised by akinesia/hypokinasia of the distal LV areas with basal normokinesia, which they named “Takotsubo syndrome” [365]. Years later, the event of Takotsubo syndrome (TS) was confirmed [366] as a syndrome caused by microvascular dysfunction resulting in temporary wall motion irregularities with a characteristic ballooning in the LV [365]. It is also referred to as stress cardiomyopathy [367] or “broken heart syndrome” [368]. It is rather a rare disease, estimated in 2% of patients with an early diagnosis of ACS [369], 90% of which are postmenopausal women with normal coronary arteries [370–372]. Major clinical indications of TS are contractile dysfunction commonly affecting the apical heart section, in the absence of coronary thrombosis [365]. Moreover, cardiac contractility unusual disruption influences 28–40% of TS patients, affecting other heart areas [373–376]. Aetiology of TS is normally physical or emotional stress, reporting 39–55% physical stress-related cases compared to 17–33% emotional stressors [377, 378]. Indeed, norepinephrine serum levels were increased in TS patients suffering from emotional stress [379]. Taken that stress prompts TS, cortisol and catecholamine levels should be investigated. In fact, TS patients’ plasma epinephrine levels were increased in the subacute phase [380]. Furthermore, an increase in norepinephrine levels was more evident in TS patients during a mental stress exam than in control subjects [381]. Also, epineprhine blood levels in TS patients were increased compared to ACS patients [382]. Further groups have reaffirmed increased levels of both norepinephrine and epinephrine in TS patients’ plasma [383], in addition to elevated cortisol levels in TS patients [384]. Besides the catecholamine apparent trigger of TS, other humour factors such as ET-1 may also be implicated in TS pathogenesis. It has been described that ET-1 causes coronary artery spasm [385–387], being the small diameter arteries the most sensitive [385]. It also contributes to microvascular myocardial dysfunction [388, 389]. Moreover, ET exerts a positive inotropic effect [390, 391] and has been seen to reduce the contractile function of isolated mouse cardiomyocytes [392]. In a study, ET-1 levels in blood plasma were twofold higher than in healthy volunteers, a signature of circulating microRNAs differentiates takotsubo cardiomyopathy from AMI; however, other investigators claim no differences in ET-1 levels in TS patients compared to their matching age, gender and risk factor group between comparable groups, as seen in a clinical study [393]. Furthermore, administration of ERA has reported an increased survival rate in heart failure rats [351, 394], suggesting ET-1 as a trigger for TS, although further research must be conducted.

## ET-1 and pregnancy complications

ET receptor expression is changing during normal pregnancy as revealed by the increased expression of ETA and ETB receptors in the uterus of pregnant versus non-pregnant women [395–397]. However, most studies addressing ET-1 functional role in BP control originated from animal studies. ET-1 supports utero-placental vasculature contractile tone, which decreases near pregnancy term [398]. Activation of ETB receptor in rats is required for optimal pregnancy outcomes [399]. During normal pregnancies, endogenous progesterone and oestrogen levels are increased, while circulating ET-1 levels are reduced [152]. ET-1 plays an important role in embryonic development, as disruption of the edn1 gene or endothelin receptor A may result in a hypomorphic pharyngeal skeleton or skeletal element fusions [12]. Endothelin ligands and receptors are exclusive to vertebrates and manage to control neural crest cell development [12].

Pregnancy-related hypertension includes many disorders such as eclampsia and preeclampsia (PE) (attenuated progesterone levels), gestational hypertension (GH) and haemolysis, elevated liver enzymes and low platelets (HELLP) syndrome [9, 11, 400]. PE is a multisystem disorder associated with increased renal vascular resistance and elevated BP, and endothelial dysfunction is one main cause of both maternal and foetal morbidity and mortality worldwide, whose underlying mechanisms are hardly understood [9, 401]. The hypoxic placenta releases anti-angiogenic factors (sFlt-1 and soluble endoglin), cell-free nucleic acids, free radicals and proinflammatory mediators and major effector ET-1 [402], which disturb the balance between endothelium-derived constricting (TXA2, angiotensin and ET-1) and relaxing factors (PGl2 and NO) [9]. These circulating cytokines stimulate further ET-1 production by EC [10]. Many groups reported increased ET-1 plasma levels of preeclamptic women in comparison to normotensive pregnancy controls [403, 404]. ET-1 umbilical vein concentrations in PE were higher than in normal pregnancies [405]. In fact, progesterone complementation to umbilical vascular EC exposed to serum from preeclamptic women weakened secretion of ET-1 in humans [406]. Other studies have shown increased plasma ET-1 levels in umbilical cord cells and renal tissues throughout late PE, suggesting that ET-1 is involved in the progression of PE rather than in the initial phases [10, 407]. Many studies have shown similar ET-1 levels in normal pregnancies and PE, presenting high levels just in severe PE and HELLP syndrome [408]. PE represents a risk for long-term diseases for mothers and their babies, such as renal, cardiovascular, CVS or neurological diseases [11].

Experimental models of PE have also revealed increased ET-1 tissue levels in both the kidney and placenta [404]. Aggarwal et al. reported a link between the elevation of sFlt-1, sEng and ET-1 in PE maternal circulation which suggests the secondary mediation of ET-1 in PE pathogenesis to these anti-angiogenic factors released by the placenta [409]. ET-1 precursor, pepro-ET-1 mRA elevated tissue levels, has also been associated with PE in experimental models. Placental ischaemic rats expressed increased pepro-ET-1 in the renal cortex and medulla as opposed to normal pregnancy animals [410]. Moreover, infusion with pro-inflammatory cytokine, TNF-α, mediated hypertension by inducing pepro-ET-1 gene expression in the placenta, kidney and maternal vasculature of pregnant rats [411]. An additional proposed mechanism for elevated ET-1 levels in PE is through activation of matrix metalloproteinase (MMP). MMP has been reported to be increased in women affected by PE and increased MMP-2 expression increased an increase in big ET-1 conversion, hence increasing vasoconstriction [412]. Another study revealed increased vascular and serum MMP-1 in women with PE, which might promote hypertension development in the mother [413]. Considerably, ETA receptor blockade enables protection against PE [414–417]. ETA receptor antagonist was infused in a rat model of PE, resulting in a rise in BP, demonstrating the key role of ET-1 [410]. Nevertheless, ETA receptors are vital for foetal development during the first trimester; thus, their use should be restricted. ETA receptor antagonists that do not transgress the placental barrier would be an alternative.

Furthermore, endothelial dysfunction, characterised by imbalances of vasoconstrictor/vasodilator factors, involves increases in blood coagulation potential (hypercoagulation) leading to uterine vessel vasoconstriction, platelet aggregation activation and resulting in final miscarriage [418]. Dubyk et al. compared ET-1 and NO serum levels in pregnancies with a risk of miscarriage, spontaneous abortion and non-developing pregnancies versus physiological pregnancies [418]. They reported a significant increase in ET-1 and decreased NO levels in all groups compared to the control group. This indicated that endothelial dysfunction was likely the cause of miscarriage in these women, serving ET-1 and NO as potential markers for endothelial dysfunction [418].

Prenatal death increases with the presence of pregnancy complications such as polyhydramnios or oligohydramnios [419, 420]. Oligohydramnios refers to a diminished volume of amniotic fluid than anticipated for gestational age which may have a foetal, placental or maternal cause or even be idiopathic [421]. Pregnancies with oligohydramnios complications might be at risk of pulmonary hypoplasia, foetal deformation or umbilical cord compression [421]. Elevated ET-1,2 concentrations were observed in pregnancies affected by oligohydramnios [422]. Moreover, studies measuring ET-1,2 levels in umbilical venous blood collected at delivery revealed increased levels in oligogydramnios infants [423]. Thus, increased levels of ET-1 in the feotus can result in oligohydramnios. A study observed lower ET-1 levels in oligohydramnios twins in respect of twins with polyhydramnios, suggesting a critical role of this hormone in amniotic fluid volume regulation [424]. No correlation between gestational age and ET-1 amniotic fluid [424] nor foetal ET-1,2 [423, 425] concentration was observed. Other studies revealed higher ET-1 levels at pregnancy term compared to at mid-trimester pregnancy [426, 427]. ET-1 might affect amniotic fluid volume regulation by causing the release of vasopressin, natriuretic peptide, aldosterone and diminishing renal perfusion [422]. Further investigations would elucidate the exact ET-1 implications in pregnancy complications.

## Multi-faceted involvement of ET-1 in migraine attacks

Migraine is a neurovascular disorder acknowledged since olden times which is greatly prevalent in society, affecting 1 in 10 people worldwide [428–430]. Migraine pathophysiology theories are very vast. Thus, whether the vasculature plays a primary role in the inception and maintenance of migraines remains uncertain [431–433]. Most studies emphasise vasodilatory mediators when researching migraines; however, vasoconstrictors and their effects must be considered. In particular, diminished levels of NO vasodilatant metabolites and an enhancement of ET-1 have been detected in migraine patients [434]*.* Studies report elevated baseline levels of ET-1 compared to control subjects [434–437]. ET-1 regulated cerebral blood flow and its receptors have been identified in the endothelium and VSMCs across the CNS and in the arterial system [70, 71]. Interestingly, plasma levels of ET-1 were found to be elevated in the early stages of migraine attacks and promptly diminished at the onset of the headache [435]. Gallai et al. also observed increased ET-1 plasma levels in the ictal phase of migraine [438]. However, contradicting data were found, revealing no significant ET-1 concentration changes during migraines [439, 440]. All studies demonstrated increased levels in venous blood during migraine attacks. Hypoxia has also been seen to increase ET-1 expression, eliciting migraine attacks with and without aura [441, 442]. One of the primary factors for migraine aura might be cortical spreading depression [443, 444], along with the cause for migraine headache [445, 446]. ET-1-induced CSD may be mediated by microinfarction on account of vasoconstriction [447]. CSD can be triggered by ischaemia or mechanical, electrical and chemical cortical stimulation [448], and has been detected in association with vascular responses throughout migraine attacks with aura [443, 449]. Moreover, migraine aura attacks are improbably linked to neuronal damage [450], as well as neuroimaging data to be clear from sublicinal infarcts or white matter *hyperintensities* [451]. Furthermore, ET-1 is related to nociception in the nervous system [452], and it is seen to induce pain and cause sensitisation to distinct nociceptive stimuli in the human peripheral nervous system [115]. It triggers the release of endogenous, migraine-inducing molecules such as NO [453] and calcitonin gene-related peptides [454], which have been shown to initiate migraine attacks in clinical studies [455]. ET-1 might influence and generate migraines with aura by originating a cascade comprising migraine-triggering substances. Broadly recognised migraine prophylaxis drugs are beta-blockers and the ACE inhibitor lisinopril which reduce both ET-1 synthesis and release in human EC [456, 457]. An AngII type 1 receptor blocker decreases ET-1 concentration in essential hypertension patients [458]. A randomised clinical trial with combined ETA/ETB receptor antagonist bosentan was ineffective in the acute treatment of migraine [459]. This study failed during migraine attacks, but ET-1 antagonists might be effective in migraine prophylaxis or when administered at the outset of attacks.

## ET-1 in ischaemic stroke

In humans, ischaemic stroke is the second leading cause of death and disability globally [460]. Based on the area and size of the brain injury, patients normally suffer from lifetime impairments, affecting from cognitive, sensory and motor to behavioural and communicative functions [461]. The majority of stroke cases results from transient or permanent obstruction of the cerebral blood vessel, depriving the brain of energy and oxygen [462]. The ischaemic cascade initiates the formation of ROS, accumulates calcium intracellularly, releases glutamate and induces inflammatory processes, resulting in tissue injury (infarction) [462]). ET-1 also induces neuronal damage [463], augments blood–brain barrier permeability and enhances vasospasm related to subarachnoid haemorrhage (SAH) [464]. ET-1 levels were seen to be elevated in both plasma and brain tissue in ischaemic stroke patients [236, 463, 465, 466]. In acute ischaemic stroke patients, plasma ET-1 levels were increased, being more marked within the initial 24 h after stroke onset, correlating to neurological damage severity [466]. Another study observed a correlation between Big ET concentrations and their specific clinical outcome (high levels: poor prognosis/low levels: more favourable outcome) [467]. ET-1, as the potent and long-lasting vasoconstrictor that it is, has been used to induce focal ischaemia in animal models, resulting in afflicted pure-motor and sensorimotor conducts which are reliant on the area of ischaemic insult in these models [468–471]. ET-1 can be applied to cortical surfaces [25], resulting in the dose-dependent ischaemic lesion with marginal ischaemic edema [468, 472], or directly onto exposed middle cerebral artery [473] as an intracranial injection [472]. Increased potential of ET-1 when delivered to conscious rats in relation to anesthesised ones has been proven [474]. Models of anterior cerebral artery occlusion and white matter ischaemia in the internal capsule have also been conducted with ET-1 [475, 476]. Furthermore, some studies utilising ET-1 to induce cerebral ischaemia models have been developed in non-human primates, in specific in marmoset monkeys [477, 478]. Its administration generated dose-dependent decreases of vessel calibre in middle cerebral arteries, succeeded by progressive reperfusion [477]. ET-1-treated marmosets displayed pronounced contralateral motor deficits in grip force [477]. Dai et al. revealed ET-1 potential to induce transient ischaemic stroke in rhesus monkeys and generate focal ischaemia in non-human primates, making it a compelling stroke and post-stroke brain repair model [479]. In fact, ET receptor antagonists have been reported to exert protective effects in animal models of stroke [93, 480, 481].

ET-1 model advantages are the low mortality rates, less invasive technique and conceivable induction of direct focal ischaemia in both superficial and deep brain areas. In contrast, the ET-1 model presents limitations related to astrocytes and neuron production of ET-1 receptor and ECE [482], which may induce astrocytosis [483]. Thus, it is suggested that ET-1 may be involved in CVS disease pathogenesis, pointing future directions towards the employment of endothelin as an early predictive factor for patients undergoing an ischaemic stroke.

## ET-1 and neurodegenerative disorders

Neurodegenerative diseases such as AD are characterised by a loss of neurons in the brain, which may result in loss of memory and cognitive function deficits [484]. There are a number of factors involved in AD neuronal degenerative alterations such as beta-amyloid deposition, pro-inflammatory cytokine/chemokine secretion and microtubule destabilisation [485]. Vascular dysfunction plays a main role in AD progression [486], and ET-1 has been described to be increased in the cerebral cortex and cerebral blood vessels in AD [39, 486, 487]. Increases in amyloid beta-protein (Aβ) indirectly stimulate ET-1 production [486, 488, 489]. Cerebral vasculature of mice infused with Aβ increased ET-1 production [490], and human neuroblastoma and brain microvascular EC exposed to Aβ increased ECE-1 and -2 secretion, resulting in elevated ET-1 secretion [39, 486, 491]. Astrocytes also regulate ET-1 expression in AD and other brain disorders [39, 40, 492]. Furthermore, increased ET-1 has been reported in a number of neurodegenerative diseases, such as multiple sclerosis, Parkinson’s disease or amyotrophic lateral sclerosis [41, 492, 493]. For instance, ALS is characterised by progressive loss of motor neurons and astrogliosis. In ALS, a neuro-inflammatory reaction takes place in glial cells, namely astrocytes and microglia [41]. Ranno et al. examined the expression of ET-1 in both spinal cords of SOD1-G93A mouse model of familiar ALS and ALS patients and reported increased ET-1 expression in both cases. In their in vitro mixed spinal cord culture model with reactive astrocytes, ET-1 exposure exerted harmful effects on MNs which were concentration and time dependent [41]. The following study investigated the underlying mechanisms of ET-1 toxic effects on MNs cultures. Their results suggest that ET-1 toxicity does not directly result from oxidative stress or COX-2 activation but requires NO and is mediated by phosphoinositide 3-kinase (PI3K) diminished pathway activation [493]. They also observed that microglia cells are not involved in ET-1 detrimental effects on MNs [493]. Thus, ET-1 signalling may be a fitting therapeutic approach to hinder MN degeneration in ALS disease.

## ET-1 in cancer

ET-1 participates in tumour growth, cell proliferation and other aspects of cancer progression in a variety of tumours [494]. In cancer types such as breast, colon, pancreatic and prostate cancer and human oral squamous carcinoma cell lines, ET-1 protein or mRNA secretion was increased [495–497]. Two distinct classifications of endothelin-derived tumours have been described. Both tumour groups hyper-secrete ET-1; however, one upregulates ETA receptors and downregulates ETB receptors slightly, such as ovarian, colon, prostate, pancreatic and renal cell carcinoma, whereas the other upregulates ETB receptors and downregulates ETA receptors, like breast and lung cancers [494]. The endothelin axis results in activation of atypical proliferation, alteration of nociceptive stimuli, apoptosis evasion, angiogenesis, cell proliferation, immune modulation and metastasis invasion, by triggering multiple signalling pathways [44]. ET-1 mitogenic activity may be increased by the interplay with growth factors such as EGF, insulin, insulin-like growth factor, TGF, PDGF, basic fibroblast growth factor and IL-6 [44].

### Ovarian carcinoma

ET-1 and ETA are overexpressed in a great number of primary and metastatic ovarian cancers, relating also with progressive stages of cancer. In fact, increased ET-1 levels were found in ascites of patients with epithelial OC, in which ETA exerts pleiotropic effects such as survival, migration and invasion [45]. Gene expression analysis studies revealed ETA as a metastasis-related gene [498] greatly expressed in post-chemotherapy specimens in relation to untreated primary ovarian carcinomas [499]. Besides ETA, EGFR is also overexpressed in OC, being usually linked to poor prognosis and related to tumour resistance to chemotherapy, thus making it a prominent therapeutic target [500, 501]. This knowledge promoted the investigation of EGFR inhibitor gefitinib (ZD1839, Iressa) along with ETA antagonist ZD4054, which revealed an enhanced efficacy, resulting in partial or complete tumour regression of ovarian carcinoma xenografts followed by decreased vascularisation, VEGF, MAPK, EGFR, matrix metalloproteinase-2 (MMP-2) and Ki-67 [502]. Kajiyama et al. observed how ovarian carcinoma cells overexpressing neutral endopeptidase (cell surface aminopeptidase that degrades ET-1 amongst other peptides) presented diminished ET-1 levels, cell proliferation, viability and invasiveness [503]. Overexpression of NEP in vivo showed reduced tumorigenesis, suggesting the use of NEP as a suppressor of ovarian carcinoma progression by targeting ET-1. In another study, patient-derived xenografts treated by dual ET-1R antagonist macitentan in combination with cisplatinum showed shutting down of the β-arr1-mediated YAP/mutp53 transcriptional programme (its activation correlates with the worst cancer prognosis) accompanied with anticancer effects in high-grade serous OC [504]. Chellini et al. (2019) evaluated signalling network for adhesion components, cytoskeletal remodelling and ECM degradation in OC. It has been concluded that the ET-1 receptor regulates extracellular matrix degradation and consequent metastatic spread in OC via β-arr1/IQGAP1 signalling pathway [46].

### Breast carcinoma

Increased expression of ET-1 and its receptors correlates with the progression of the disease and its malign potential [505]. Also, ECE-1 overexpression in breast cancers has been linked to adverse outcomes [506], whereas increased expression of ETA relates to low disease-free survival and overall survival. Increased ETA expression in breast cancer relates to chemotherapy resistance, thus serving ETA as a predictive marker for chemotherapy therapy outcomes [505]. Overexpression of ET-1 has been detected in numerous diseases [20]; indeed, in breast carcinoma, its increased expression has been associated with poor disease prognosis [507]. High levels of blood ET-1 result in overall hypoxic effects, which consecutively lead to metastatic disorders [47, 48, 508]. Additionally, ET-1 blood patterns are not well understood in regard to the distinct breast cancer subtypes.

A recent work reported increased circulating levels of ET-1 in breast cancer also correlated to LV remodelling in this patient cohort compared to healthy controls [509]. In vitro studies have reported a correlation of ET-1 axis expression levels and breast cancer cell line invading potential [510]. Grimshaw et al. established an in vitro invasive breast tumour cell phenotype by exposing tumour cells to ET-1, through the action of ETA and ETB receptors and elevated MMP activity [511]. In a model of breast carcinoma, the use of dual endothelin receptor antagonist (ERA), bosentan, inhibited bone metastasis, as well as tumour growth and vascularisation [512]. Another study reported high relevance of the ETB receptor in breast carcinogenesis. Silencing the EDNRB gene modulates invasiveness in breast cancer cells towards ET-3. ETB receptor isoform specific controls breast cancer cell invasiveness and isoform- and subtype-specific dissimilarities in patient survival [513]. Consequently, knockdown of the ETB receptor by a specific shRNA significantly suppresses the proliferation and metastatic parameters in MDA-MB-231 and BT549 cells and upregulates apoptosis [49]. In consensus, suppression of ETB receptor expression inhibited cancer growth in the mice xenograft model.

### Prostate carcinoma

The ET axis has been described to contribute to prostate cancer pathophysiology. ET-1 in healthy prostate subjects is produced by epithelial cells and maximum levels are detected in seminal fluid. In prostate carcinoma, ET-1 local levels are increased due to a decrease in ET-1 clearance pathway constituents [514]. ETA increased expression is seen in primary and metastatic prostate carcinomas, correlating with tumour development and grade, where ET-1 exerts cancer progression actions. In a study where ETA antagonist atrasentan was administered to prostate cancer patients, they revealed a reduction in pain and prostate cancer progression biomarkers [515]. Another ETA orally active antagonist has been researched in phase II clinical trial, revealing general improvement when administered to metastatic hormone-refractory prostate cancer patients [516]. Silencing ET-1 by RNAi significantly suppresses the progression and invasion of PC-3 prostate cancer cells. These effects affect several signalling pathways including Erk1/2/Bcl-2/Caspase-3, PI3K/Akt/Caspase-3 and MMPs (MMP-2 and MMP-9) [517]. To this end, the role of the proteasome inhibitor bortezomib in ET-1 was evaluated, observing an induction of cell signalling in prostate cancer. ET-1 supplement to PC-3 cells decreased Bad, p53, p21 and p27 expression, and increased IL-8, VEGF, proteasomal activity and NFkappaB levels. Furthermore, these pro-neoplastic effects of ET-1 in PC-3 cells were reversed by the treatment with bortezomib which suggests its distinct role in the regulation of cancer cell proliferation and apoptosis [518].

### Colon carcinoma

ET-1, ETA and ECE-1 are actively expressed in colon adenocarcinoma cells in comparison to non-cancerous cells [519]. ET-1 expression is elevated in the majority of primary human colon cancers [520]. Moreover, ET-1 by inhibiting β-catenin signalling is capable of recovering colon cancer cells from growth arrest and apoptosis, revealing ET-1 oncogenic function in colon carcinoma [520]. In vitro studies revealed upregulation of ETA expression in all cell sorts compared to healthy colon cells and described ET-1 as a mitogen for colorectal cancer cells. Cancer-associated blood vessels and fibroblasts revealed increased ETA binding while having downregulated ETB, which prevailed in the non-cancerous colon [519]. This shift might induce ETA-related ET-1 colorectal cancer growth and neovascularisation, suggesting the benefit of ETA receptor antagonist as colon carcinoma adjunct therapy [521]. Wang et al. [522] reported that the ETA receptor stimulates colon tumorigenesis via an increase in cell proliferation and migration. Mechanistic evaluation of this effect revealed ET-1-induced YAP/TAZ dephosphorylation and transcriptional activation in multiple colon cancer cells. In detail, ETA receptor activation stimulates G protein Gαq/11 and Rho GTPase pathway and suppresses the Hippo pathway that collectively leads to ETA receptor-induced carcinogenesis via YAP/TAZ activation. The role of the ET axis and its downstream signalling in metastatic colon carcinogenesis was investigated in a clinical study [50]. The authors evaluated the mRNA expression of 36 genes linked with the ET axis using 18 non-metastatic and 20 metastatic colon carcinomas in comparison with normal colon mucosa. Data showed that the majority of genes in the ET axis are overexpressed (17 from 36) in malignant colon tissue. It has been concluded that suppression of PTEN expression may boost a malignant phenotype in colon carcinogenesis.

### Other cancers

ET-1 has also been detected in the lungs, cervix, colon, melanoma, pancreatic, glioblastoma, neuroblastoma and bladder cancers. In this regard, the ETB receptor represents a potential prognostic marker for lung adenocarcinoma patients. ETB receptor may act via regulation of the ERK signalling pathway in lung adenocarcinoma [523]. In aggressive cervical squamous cell carcinoma, overexpressed ET-1 contributes to the angiogenesis signalling pathways [43]. Clinical data indicate that a MAPK pathway inhibitors combined with ETB receptor antagonists could have a synergistic anticancer effect in melanoma patients with upregulated MAPK signalling [524]. It has been documented that ET-1 stimulates MAP kinase and AP-1 signalling pathways, increasing the expression of MMP-9 and MMP-13 and consequently activating cell migration in human glioblastoma [525]. Pancreatic ductal adenocarcinoma  is related to increased levels of ET-1 [526, 527], revealing likewise overexpression of ET axis components such as ETB, in PDAC tissues [528]. A recent study observed the expression of ET axis constituents in pancreatic acinar and islet cells compared to the minor levels in pancreatic ducts of control mice [298].

Many investigations were carried out with ET-1 receptor antagonists, range from preclinical to clinical trials reaching phases II and III with either selective or specific ETA or dual ETA/ETB antagonists or even selective ETB agonists IRL-1620 [297, 529, 530]. ERAs’ action is exerted firstly on ET-1, which in turn blocks autocrine and paracrine pathways, hampering tumour growth and angiogenic effects, making them promising tools for cancer therapeutics [531]. IRL-1620 may be employed to enhance drug delivery (in chemotherapy and radiation therapy) due to the increase in tumour perfusion which leads to enhanced therapeutical effectiveness [297, 529]. Given that ETA-specific antagonists in cancer have portrayed unfavourable results, a potential combination of ETA/ETB blockage with macitentan may be advisable for cancer, as observed in preclinical studies [297]. In fact, the concomitant enhancement of antimumoral immune reactions and suppression of tumour cell invasiveness would make macitentan a strong candidate for cancer therapy [297]. It is critical to comprehend the entire range of molecules and pathways activated by ET-1 in order to design targeted therapies. Additionally, investigating novel combination strategies by merging macitentan with other molecular target therapies, chemotherapy or radiation therapy is required.

## ET-1 and individual COVID-19 outcomes

The severe acute respiratory syndrome coronavirus 2 (SARS-CoV-2) poses a hazard to individuals with chronic health afflictions which are more prone to progress to a life-menacing stage characterised by inflammatory lung processes and cytokine outbursts [61].

Recent findings hypothesise receptor-interacting protein kinase 3 [532] oligomerisation of open reading frame (ORF)-3a to drive necrotic cell death, leading to tissue injury and detrimental inflammation [60]. More precisely, ET-1 production in the dendritic cell has been seen to be stimulated by necroptosis (regulated necrosis) [533]. Abdul et al. described that blockage of ET receptors with bosentan impaired the necroptosis pathway along with increased brain microvascular endothelial cell migration, proposing that ET-1 activates programmed cell death pathways under inflammatory stimuli [533].

As previously noted, ET-1 detrimental action is exerted via ETA present on pulmonary smooth muscle cells and ETB in vascular wall muscle cells which are upregulated in the context of systemic and pulmonary hypertension [534]. Thus, in this context, ERAs have been validated to be used for treating cases of pulmonary arterial hypertension (PAH) [62]. This is the case of bosentan, known for decreasing profibrotic and pro-inflammatory cytokines (interleukin (IL)-2, -6, -8 and interferon-γ) in patients with scleroderma [535]. Furthermore, bosentan has proven efficacy against some viruses together with exerting anti-IL-6 effects [536, 537]. In a case report of influenza A-related acute respiratory distress syndrome, bosentan administration led to symptom amelioration along with mechanical ventilation weaning [538]. Furthermore, other studies have also used ERAs as cancer therapeutic drugs, diminishing the effect of ET-1 on tumour progression, by constraining epidermal growth factor receptor and blocking angiogenic effects [531]. Also, ETA receptor antagonists like ERAs have been used for the treatment of chronic kidney diseases along with hypertension by opposing ET-1 vasoconstricting effects [72, 531]. These results suggested a potential use of ERAs as a drug against SARS-CoV-2 inflicted hypertension and hypoxic vasoconstriction [63]. However, further tools for improved identification, intervention and prognosis of COVID-19 patients are needed, along with further research on potentially involved mechanisms.

## ET-1 as the target in the framework of 3P medicine: status quo and outlook

Depending on the pathway, the level of release and corresponding targets, ET-1 is involved in the regulation of physical and mental well-being; female and male health; modulation of senses, pain and stress reactions; drug sensitivity; healing processes; amongst others.

### Endothelin in disease prediction

Shifted ET-1 homeostasis may influence and predict development and progression of suboptimal health conditions, metabolic impairments with cascading complications, ageing and related pathologies, CVD, neurodegenerative pathologies and aggressive subtypes of cancer, such as metastasing breast and prostate malignancies, thereby modulating individual outcomes of both non-communicable and infectious diseases such as COVID-19 with versus without severe complications.

### Endothelin in disease prevention early in life

The most prominent examples are vascular dysregulation, systemic vasoconstriction and arterial stiffness linked to elevated ET-1 levels, which individually may lead to cascading pathologies discussed in this article. Physical training has proven to decrease BP and enhance endothelial function in hypertensive and healthy subjects [32, 539] and it has improved arterial stiffness by elevating vasodilation/NO availability and reducing vasoconstriction/ET-1 level in adults [540]. Aerobic exercise reduces ROS and increases NO bioavailability improving the balance between NO and ET-1 [2]. Thereby, DNA methylation of EDN1, NOS2, ALU and TNF genes is crucial in reducing systemic BP, meanwhile implementing continuous aerobic physical training [2]. A study which examined the effect of combined resistance and aerobic exercise (CRAE) training in obese pre-hypertensive girls on BP, body composition, blood nitrite/nitrate (circulating NO marker) and ET-1 levels [1] has demonstrated that CRAE training managed to reduce SBP, arterial stiffness, increased total nitrite/nitrate and decreased ET-1 levels and decreased percentage of body fat and increased lean body mass in obese adolescent girls with pre-hypertension. These results convey that CRAE-mediated decrease in BP is possibly due to an improvement in vascular endothelial cell function, as evidenced by the elevated blood nitrite/nitrate and diminished ET-1 levels, and would be a potential therapeutic treatment for high BP, preventing prospective CVD [1]. In consensus, other studies demonstrated how physical training decreases ET-1 and increases NO circulating levels collectively leading to reduced BP [541, 542]. To this end, increased vasodilatation by the NO bioavailability reduces arterial stiffness through a structural alteration of the endothelium that in turn reduces BP in pre-hypertensive patients [1].

### Endothelin in the prevention of accelerated ageing

Approaching disease prevention must be the initial phase to achieve a healthy/physiologic ageing [177]. Endothelin activation along with inflammatory processes is related to a greater incidence of chronic diseases in the elderly compared to the younger population [543]. Thus, endothelial therapy and physical exercise are preventive procedures enabling a favourable ageing process [91]. Physical activity has been shown to have comparable effects as drug treatments [544], since it enhances the release and bioactivity of endothelial NO, which in turn reduces the production and biological activity of ET-1 [177, 545, 546]. Some other related effects are anti-hypertensive, anti-inflammatory and anti-diabetic functions [547, 548]. The term endothelial therapy comprises preventive efforts, physical training and potential use of endothelial factor treatment in an effort to improve standard endothelial homeostasis [177, 549, 550]. In fact, elderly women exposed to endurance training presented reduced ET-1 circulating levels [205]. Training also dampens ET-1 vascular tone rise in the elderly [179, 551], along with a suppressed age-related BP increase [545, 546]. Indeed, there is a range of studies assessing ET-1-induced disease prevention, and ERAs have specifically been studied to prevent severe disease progression, delay disease onset or as potential therapeutics. In fact, ETA-specific ambrisentan has been proven to be advantageous in preventing vasoconstriction and cellular proliferation mediated by VSMC ETA receptors, concomitantly maintaining ETB receptor vasodilator function [552, 553]. Macitentan as dual ERA inhibits ET-1 binding to both ETA and ETB, differing from bosentan and ambrisentan in its slow receptor dissociation characteristics at a cellular level [554, 555]. It appears that macitentan is able to block ET-1-promoted signalling in a more effective manner than other ERA [556]. ERA may be used for the prevention of fibrotic disease development. In fact, bosentan is known to inhibit ECM deposition and ET-1-induced fibroblast proliferation, besides being able to reduce pulmonary, cardiac, hepatic and renal fibrosis in many disease models by endothelin axis activation [557]. The hepatic fibrogenic response was also diminished in liver disease experimental models by inhibiting endothelin signalling with ERA [284, 285]. Furthermore, ET-1 is believed to be an essential intermediary in cerebral vasospasm subsequent to a SAH, given that animal and human studies report increased blood and cerebrospinal fluid ET-1 levels [558]. ET-1 and NO interplay is crucial in preserving cerebral vascular dilation and cerebral blood flow upon SAH. Vasospasm can be prevented by either ET-1 antagonist or ECE inhibitor administration, being ETA receptor antagonist clazosentan favourable in preventing or reversing cerebral vasospasm [559]. In SAH experimental animal models, clazosentan has been seen to decrease or reverse cerebral vasospam [560]. Further reports in experimental SAH rabbits, rats, dogs and monkey models have proven the prevention or reduction of vasospam following endothelin antagonist administration [561]. Despite ERAs are a promising therapeutical treatment for many diseases, additional studies are required to assess the benefits and safety of ERA treatment along with the validity of combination therapy schemes.

### Endothelin, targeted therapy and personalised medicine

Since ET-1 discovery, the therapeutic strategy has evolved around the application of either ETA receptor (ambrisentan) or ETA/ETB dual blockage (bosentan and macitentan) by using orally administered small molecules antagonists [562]. There has been recent advancement in this field with the use of selective peptide agonists and antagonists along with monoclonal antibody antagonists, potentially broadening therapeutical extent to further pathophysiological settings. The use of ETA antagonist ambrisentan together with the phosphodiesterase (PDE) 5 inhibitor tadalafil was proven to enhance PAH treatment [563]. Combining two vasoconstrictive sites such as angiotensin AT1 and ETA antagonist, sparsentan has become another innovative approach [564]. In addition, single-nucleotide polymorphism (SNP) has been studied to validate its genome implication, as it is related to numerous vascular diseases where elevated big ET precursor plasma levels have been identified [565]. This SNP would potentially enable patient stratification for assignation to ET treatment and allow for a more personalised therapy [565].

#### Monoclonal antibodies (MABs)

MABs have been widely researched as they are extremely selective for their targeted protein and present an extended plasma half-life [566]. Although manufacturing costs of MABs are quite substantial, they provide a broader range of treatment approaches compared to small molecules [567]. ETB receptor MAB, rendomab-B1 (RB1), was more effective than BQ788 competing for ET-1 [568]. RB1 was tested in melanoma cells, exerting low affinity for ETB receptors displayed in this cancer, which suggests diversity in ETB tumour subtypes [56]. A novel MAB, rendomab-B4 (RB4), was reported to attach to ETB receptor from three distinct melanoma cell lines but not to ETB on human embryonic kidney or native receptors in human umbilical vein endothelial cells [56]. Thus, RB1 and –B4 binding qualities present disparities that may be related to post-translational modifications [569]. ETA receptor MAB, rendomab-A63 (RA63), presents a sub-nanomolar binding affinity, while not being modified by an excess of ET-1 [570]. RA63 also binds ETA present on glioma stem cell surface [571]. As a result of MAB increased half-life, they may be a suitable therapeutic scheme considering ET-1 sustained vasoconstrictor activity, which has been suggested to bind irreversibly to ETA receptors. However, a study revealed that ET-1 binding cleavage from cloned ETA receptors although slow compared to further vasoactive elements was not irreversible [572]. ET-1 prolonged effect could be reversed both in vivo and in vitro by small-molecule antagonists [573, 574].

#### Dual AT1/ETA receptor antagonist

Preclinical studies demonstrated how the combination of angiotensin AT1 and ETA receptor antagonist blockage resulted in more effective in reducing BP in hypertension animal models than each antagonist alone [575]. Thus, sparsentan (BMS-346567) is an orally active dual AT1/ETA antagonist, which presents a similar increased affinity for both receptors. Preclinical data seem to indicate an effective blockage of direct vascular actions of both vasoconstricting molecules; however, their effects on downstream signalling and side effects comparison are not yet apparent [576]. Phase II trials with sparsentan are being conducted to evaluate focal segmental glomerulosclerosis treatment [577].

#### ETA antagonist and PDE5 inhibitor combination

Usually, monotherapy utilising ET receptor antagonists or PDE5 inhibitors is standard when treating PAH patients [578]; however, preclinical studies have revealed that a dual combination may be advantagenous [579]. A murine study stated a mitigation of ET-1 constricted isolated rat pulmonary arteries after treatment with ETA antagonist ambrisentan and PDE5 inhibitor tadalafil [579]. Translational studies reported a reduced risk of clinical-failure events when compared to either monotherapy treatment [563, 580]. Nevertheless, increased frequency of undesirable adverse effects appeared with the inhibitor combination therapy, possibly being related to ETB receptor activation [581].

The aim of personalised medicine is to adjust drug therapies and individualise patient treatment. SNP detection may result in an association between the patients’ genetics and a target against the presumed disease trigger. Gupta et al. determined an ordinary A/G SNP (rs9349379) in the Phosphatase And Actin Regulator 1 (PHACTR1) gene, which regulated edn1 gene expression of ET-1 along with being involved in endothelial cell survival and tubule formation [565]. They speculated that big ET-1 amounts would be more elevated in patients with the minor and most common allele (G/G) [582], intermediate in those with A/G and minimal with A/A at rs9349379, and revealed a considerable relation between G genotype and an increase of 20% in plasma levels. Thus, individuals with G/G allele would present increased ET-1 levels, leading to enhanced vasoconstriction and contributing to angina and vasospasm in coronary artery disease patients [565]. Furthermore, G/G SNP allele patients are more susceptible to ETA antagonists than A/G or A/A. Thus, as demonstrated, SNP testing would be valuable in allowing patient stratification to allocate them with the optimal therapy and personalising endothelin treatment within this field.

**Fig. 1:**
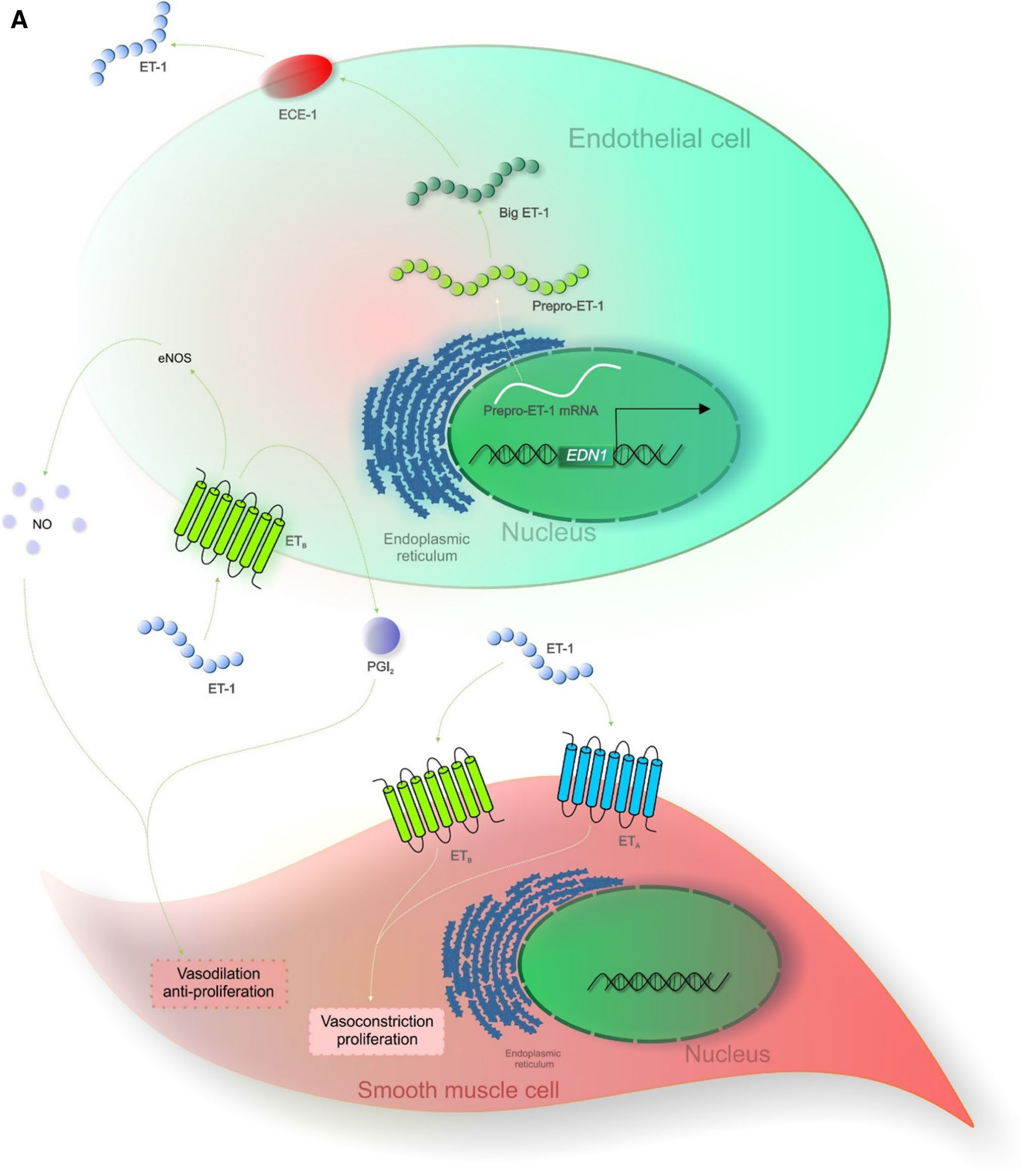

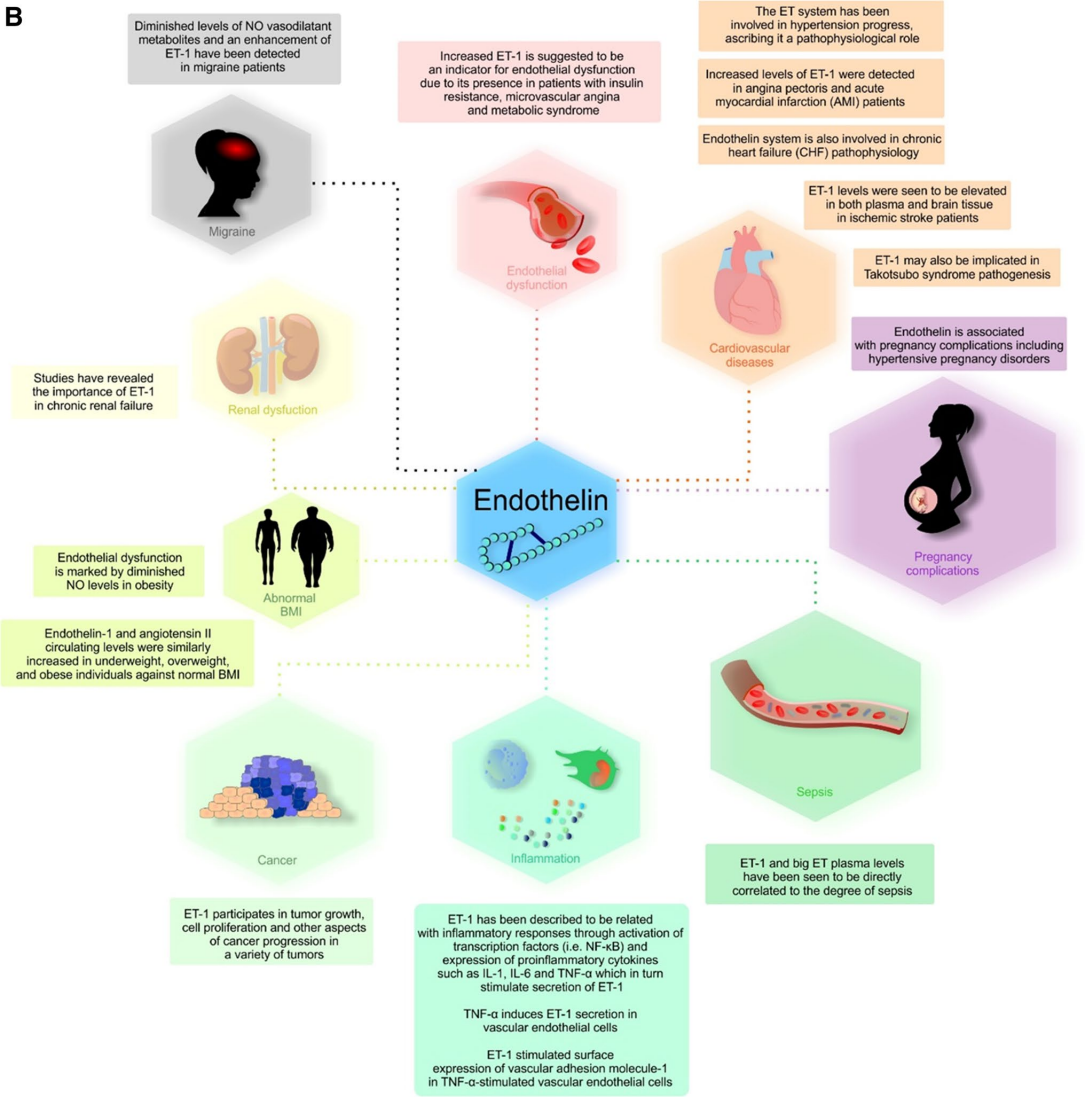
**A** Schematic presentation of the endothelin-1 production and functionality. Abbreviations: ETA, endothelin A receptor; ETB, endothelin B receptor; ET-1, endothelin-1; PGI2, prostacyclin; eNOS, endothelial nitric oxide synthase, ECE-1, endothelin-converting enzyme 1; EDN1, endothelin 1 gene; NO, nitric oxide. **B** Endothelin-1 involvement into a spectrum of pathophysiological processes and disorders

The involvement of endothelin-1 in physiologic processes and diseases relevant for predictive diagnostics targeted prevention and personalisation of treatment algorithms are summarised in Fig. 1A and B.

## Data Availability

Not applicable.

## Abbreviations

5-HAT: Serotonin
ACS: Acute coronary syndrome (ACS)
AD: Alzheimer’s disease
AMD: Age-related macular degeneration
AMI: Acute myocardial infarction
AngII: Angiotensin II
BP: Blood pressure
CHF: Congestive heart failure
CNS: Central nervous system
COX: Cyclooxygenase
CRAE: Combined resistance and aerobic exercise
CVD: Cardiovascular disease
CVS: Cerebrovascular
DOCA: Deoxycorticosterone acetate
E2: Oestradiol
EAH: Essential arterial hypertension
EC: Endothelial cells
ECE: Endothelin-converting enzyme
EDD: Endothelium-dependent dilation
edn1: Gene encoding ET-1
ERAs: Endothelin receptor antagonists
ET-1: Endothelin-1
ET-2: Endothelin-2
ET-3: Endothelin-3
ETA: Endothelin A receptor
ETB: Endothelin B receptor
HELLP syndrome: Haemolysis, elevated liver enzymes and low platelets syndrome
i.c.v: Intracerebroventricularly
IL: Interleukin
LV: Left ventricle
MABs: Monoclonal antibodies
MMP: Metalloproteinase
MMP-2: Metalloproteinase-2
NO: Nitric oxide
NOS: NO synthase
OC: Ovarian cancer
OM: Olfactory mucosa
ORF: Oligomerisation of open reading frame
PAH: Pulmonary arterial hypertension
PDE: Phosphodiesterase
PDGF: Platelet-derived growth factor
PE: Preeclampsia
PG: Prostaglandin
PGE-2: Prostaglandin E2
PGl2: Prostacyclin
PH: Paedriatic hypertension
PHACTR1: Phosphatase and actin regulator 1
PPH: Paediatric pre-hypertension
Prepro-ET-2: Peproendothelin-2
Prepro-ET-3: Peproendothelin-3
RA63: Rendomab-A63
RB1: Rendomab-B1
RB4: Rendomab-B4
ROS: Reactive oxygen species
SAH: Subarachnoid haemorrhage
SARS-CoV-2: Severe acute respiratory syndrome coronavirus 2
SBP: Systolic blood pressure
SCD: Sickle cell Disease
SNP: Single-nucleotide polymorphism
TGF-β: Transforming growth factor-beta
TNF-α: Tumour necrosis factor-alpha
TS: Takotsubo syndrome
VCAM-1: Vascular adhesion molecule-1
VSMCs: Vascular smooth muscle cells
VSMCs: Vascular smooth muscle cells
vWF: Von Willebrand factor (vWF)
WH: Wound healing
WKY rats: Wistar–Kyoto rats
YAP: Yes-associated protein
